# Delayed Recognition of Duchenne Muscular Dystrophy During an Acute Respiratory Illness in an 11-Year-Old Boy: A Case Report

**DOI:** 10.7759/cureus.117664

**Published:** 2026-10-09

**Authors:** Momna Ahsan, Esha Sharma, Munazza Iqbal, Rabia Azhar, Saad Jamil

**Affiliations:** 1 Gastroenterology and Hepatology, University Hospitals Birmingham NHS Foundation Trust, Birmingham, GBR; 2 Internal Medicine, Wroclaw Medical University, Wrocław, POL; 3 Internal Medicine, University of Arizona College of Medicine - Tucson, Tucson, USA; 4 Internal Medicine, Lahore Medical and Dental College, Lahore, PAK; 5 Internal Medicine, Allama Iqbal Medical College, Lahore, PAK

**Keywords:** case report, diagnostic delay, duchenne muscular dystrophy, lower respiratory tract infection, proximal muscle weakness

## Abstract

Duchenne muscular dystrophy (DMD) is a progressive X-linked dystrophinopathy that typically becomes clinically apparent in early childhood, although recognition may be delayed when longstanding motor abnormalities remain uninvestigated. We report a case of an 11-year-old boy who presented with fever, productive cough, mild respiratory distress, and a peripheral oxygen saturation of 88%-90% on room air. Further history revealed delayed motor milestones and progressive lower-extremity weakness over several months. Neurological examination demonstrated predominantly proximal weakness of the lower extremities, hypotonia, diminished reflexes, calf pseudohypertrophy, and a Gowers maneuver when rising. Laboratory evaluation showed neutrophilic leukocytosis, an elevated C-reactive protein level, and a serum creatine kinase (CK) concentration of 8,500 U/L. Mild elevations in aspartate and alanine aminotransferases were considered compatible with skeletal muscle injury. Molecular testing identified a pathogenic hemizygous deletion of exons 45-50 of the *DMD* gene, establishing the diagnosis of DMD without the need for muscle biopsy. The concurrent respiratory illness was treated with supplemental oxygen, empiric intravenous antibiotics, and supportive care. The patient was subsequently referred for multidisciplinary neuromuscular management and genetic counseling. This case emphasizes the importance of evaluating acute and chronic findings in parallel. In boys with delayed motor development, progressive proximal weakness, or calf pseudohypertrophy, a focused neuromuscular examination and CK measurement may facilitate timely recognition and molecular confirmation of an underlying dystrophinopathy despite a competing acute presentation.

## Introduction

Duchenne muscular dystrophy (DMD) is an X-linked dystrophinopathy caused by pathogenic variants in the *DMD *gene and characterized by progressive skeletal muscle degeneration with subsequent cardiac and respiratory involvement. Clinical manifestations usually become apparent in early childhood and may include delayed motor milestones, difficulty running or climbing stairs, frequent falls, Gowers sign, and calf pseudohypertrophy [1,2]. Despite this recognizable phenotype, diagnosis may be delayed when early motor abnormalities are subtle, gradually progressive, or insufficiently investigated. Timely recognition is clinically important because molecular confirmation facilitates consideration of corticosteroid therapy, cardiac and respiratory surveillance, rehabilitation, genetic counseling, carrier assessment, and evaluation for genotype-specific therapies when available [2,3].

An acute illness may bring an undiagnosed neuromuscular disorder to clinical attention; however, its immediate manifestations may also divert attention from a preexisting pattern of motor dysfunction. Careful distinction between acute findings and longstanding symptoms is therefore essential. We report a case of an 11-year-old boy evaluated during an acute hypoxemic respiratory illness whose history of delayed motor milestones and progressive lower-extremity weakness, together with calf pseudohypertrophy and a markedly elevated serum creatine kinase (CK) level, prompted molecular testing. Detection of a pathogenic hemizygous deletion of exons 45-50 of the *DMD *gene established the diagnosis of DMD. This report illustrates the clinical reasoning that led from an acute respiratory presentation to the recognition of an underlying dystrophinopathy and emphasizes the value of focused neuromuscular assessment and phenotype-directed genetic testing in preventing further diagnostic delay.

## Case presentation

An 11-year-old boy presented with progressive bilateral lower-extremity weakness accompanied by an acute febrile respiratory illness. His parents reported that the weakness had progressed over several months and was most apparent when he attempted to rise from a seated position. They had also noticed bilateral calf enlargement, and his mother recalled a history of delayed motor milestones. He had not previously undergone a formal neuromuscular evaluation. The acute illness began with a productive cough lasting seven days, accompanied by small amounts of yellow sputum. He developed fever with chills one day before presentation, which partially responded to antipyretic medication.

On examination, the patient was thin and in mild respiratory distress. He was febrile, with a heart rate of 110 beats/min, a respiratory rate of 22 breaths/min, and a peripheral oxygen saturation of 88%-90% on room air. A dry, intensely pruritic, discolored skin eruption was present and was considered clinically compatible with atopic dermatitis, although no formal severity assessment was performed. Respiratory examination revealed bilateral vesicular breath sounds with end-inspiratory crackles. Cardiovascular and abdominal examinations were unremarkable.

Neurological examination showed preserved muscle bulk and tone in the upper extremities, with grade 5/5 strength as per Medical Research Council and intact deep tendon reflexes [4]. The lower extremities exhibited hypotonia, marked symmetric proximal weakness, and diminished deep tendon reflexes. Plantar responses were flexor bilaterally. Both calves were visibly enlarged and firm on palpation, consistent with calf pseudohypertrophy (Figure 1). When rising from a seated position, the patient leaned forward and used both upper extremities for support, demonstrating a Gowers maneuver indicative of clinically significant proximal weakness. The combination of delayed motor development, progressive proximal weakness, calf pseudohypertrophy, and a Gowers maneuver raised suspicion of an inherited muscular dystrophy. The principal differential diagnoses included a dystrophinopathy, such as Duchenne or Becker muscular dystrophy, another limb-girdle muscular dystrophy, and an inflammatory myopathy.

**Figure 1:**
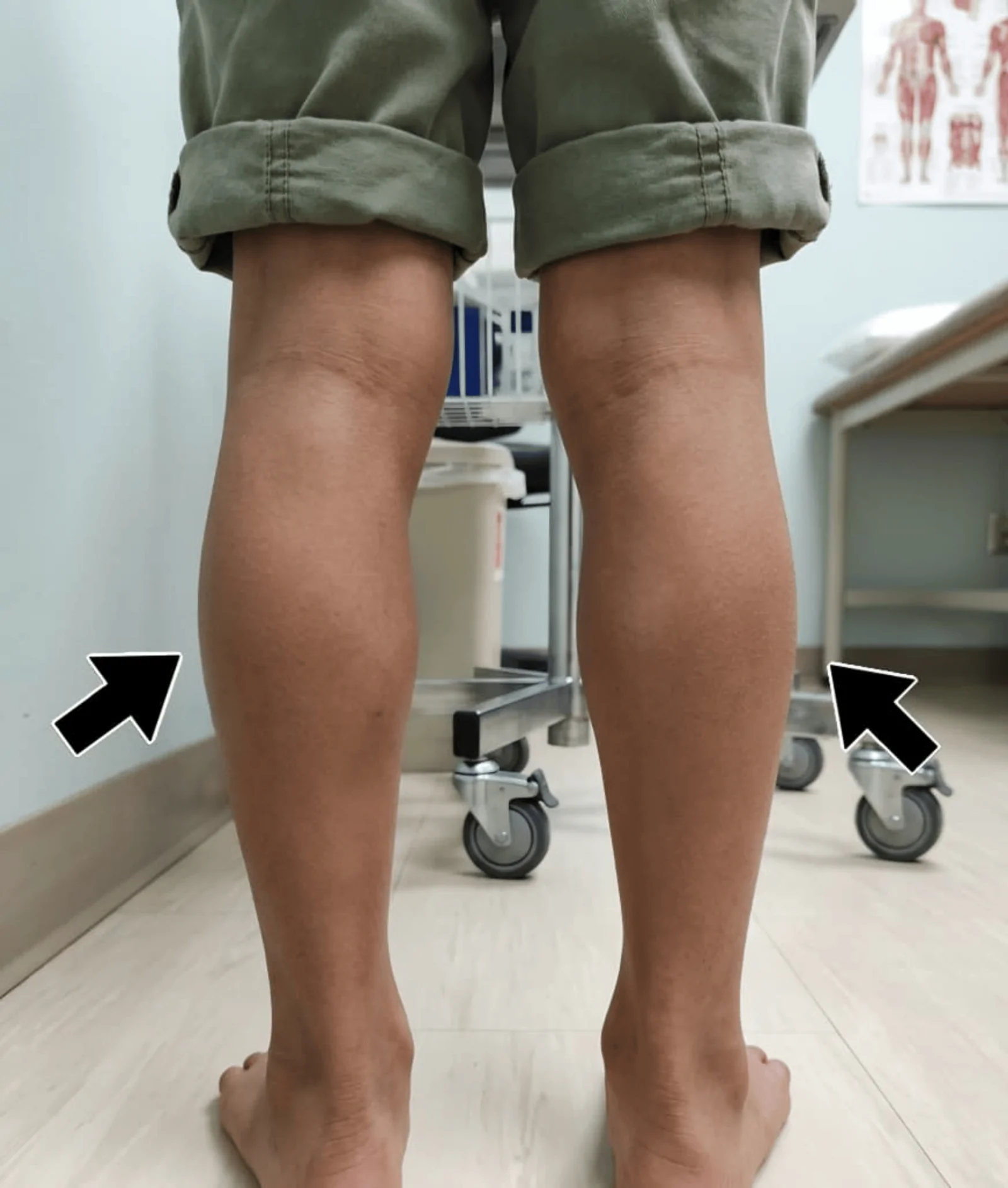
Bilateral calf pseudohypertrophy, indicated by the arrows

Laboratory evaluation demonstrated marked neutrophilic leukocytosis and an elevated C-reactive protein level, supporting a concurrent acute inflammatory or infectious process. The serum CK level was markedly elevated at 8,500 U/L, approximately 34 times the upper reference limit. Although this finding was not specific to DMD in isolation, its concordance with the neuromuscular phenotype substantially increased suspicion of muscular dystrophy. Aspartate aminotransferase and alanine aminotransferase levels were mildly elevated, whereas bilirubin and alkaline phosphatase levels were within their respective reference ranges. This pattern suggested muscle-derived enzyme release rather than isolated hepatobiliary disease. Renal function, coagulation parameters, serum electrolyte levels, hemoglobin, and platelet count were within their respective reference ranges. Selected laboratory findings are presented in Table 1.

**Table 1:** Selected laboratory findings at presentation g/dL, grams per deciliter; mg/dL, milligrams per deciliter; mm/h, millimeters per hour; mmol/L, millimoles per liter; U/L, units per liter.

Investigation	Result	Reference interval
White-cell count	28.4 × 10⁹/L	4.0–11.0 × 10⁹/L
Neutrophils	83%	40%–80%
Lymphocytes	10%	20%–40%
Hemoglobin	15.1 g/dL	13.0–18.0 g/dL
Platelets	178 × 10⁹/L	150–400 × 10⁹/L
C-reactive protein	66	<5
Erythrocyte sedimentation rate	20 mm/first hour	0–25 mm/first hour
Creatine kinase	8,500 U/L	40–250 U/L
Aspartate aminotransferase	50 U/L	≤40 U/L
Alanine aminotransferase	55 U/L	≤40 U/L
Alkaline phosphatase	90 U/L	40–120 U/L
Total bilirubin	0.279 mg/dL	0.3–1.2 mg/dL
Urea	35 mg/dL	10–50 mg/dL
Creatinine	0.7 mg/dL	0.5–0.9 mg/dL
Sodium	138 mmol/L	135–145 mmol/L
Potassium	4.1 mmol/L	3.5–5.0 mmol/L

The respiratory findings, including hypoxemia, fever, productive cough, neutrophilic leukocytosis, and an elevated C-reactive protein level, were suggestive of a concurrent lower respiratory tract infection. Supplemental oxygen was administered, and empiric intravenous broad-spectrum antibiotic therapy was initiated. Chest physical therapy, gentle range-of-motion exercises, nutritional support, and cardiopulmonary monitoring were also provided. The acute respiratory illness was managed separately from the longstanding motor abnormalities.

Molecular testing was subsequently performed. Multiplex ligation-dependent probe amplification (MLPA) for deletion and duplication analysis of the *DMD *gene, followed by confirmatory next-generation sequencing, identified a hemizygous deletion of exons 45-50 at Xp21.2. The deletion was classified as pathogenic and predicted to disrupt the open reading frame. No additional pathogenic sequence variants or duplications were identified. In conjunction with the characteristic clinical phenotype of progressive proximal weakness, calf pseudohypertrophy, and a Gowers maneuver, supported by the markedly elevated CK level, the pathogenic hemizygous deletion of exons 45-50 established the diagnosis of DMD without requiring a muscle biopsy.

Following stabilization of the respiratory illness and molecular confirmation of DMD, the patient was referred for multidisciplinary evaluation by pediatric neurology, cardiology, pulmonology, rehabilitation medicine, and genetic counseling services. The proposed long-term management plan included corticosteroid therapy, regular physical therapy and stretching, nutritional support, and cardiac and respiratory surveillance through electrocardiography, echocardiography, and pulmonary function testing. Genetic counseling was provided to the family. Because the deletion of exons 45-50 may be amenable to exon 51-skipping therapy, assessment for mutation-specific treatment was recommended, with eligibility to be determined according to the applicable regulatory criteria, clinical status, and treatment availability.

## Discussion

DMD usually becomes clinically apparent in early childhood through delayed motor development, frequent falls, difficulty running or climbing stairs, calf pseudohypertrophy, and use of a Gowers maneuver [1]. Nevertheless, population-based surveillance studies have consistently demonstrated a substantial interval between the first recognized manifestations and definitive diagnosis. In a contemporary cohort, the mean ages at the first documented signs and diagnostic confirmation were 2.7 and 4.9 years, respectively [5]. Earlier surveillance data similarly identified a diagnostic delay of approximately 2.5 years, representing missed opportunities for timely genetic counseling, initiation of corticosteroid therapy, and appropriate clinical surveillance [6]. Diagnosis at 11 years represents delayed recognition relative to the ages at which clinical manifestations and diagnostic confirmation commonly occur. Progressive motor impairment and disease-related complications may be present by this age, warranting comprehensive assessment. In the present case, progressive weakness was documented, but cardiac and chronic respiratory complications were not established. The history of delayed motor milestones and progressive weakness indicates that the disorder preceded the acute admission. However, the limited early developmental history prevents precise determination of symptom onset or identification of specific missed diagnostic opportunities. The presentation is therefore best interpreted as delayed recognition of DMD rather than late-onset disease.

The acute respiratory illness provided the clinical setting in which the chronic motor phenotype was recognized but did not adequately explain it. Fever, productive cough, hypoxemia, crackles, neutrophilic leukocytosis, and an elevated C-reactive protein level were consistent with a concurrent lower respiratory tract infection rather than an atypical manifestation of DMD. Similarly, the pruritic skin eruption was considered clinically compatible with atopic dermatitis, but no evidence supported a causal relationship with the dystrophinopathy. The principal educational feature of this case is the coexistence of an acute illness and a previously unrecognized chronic neuromuscular disorder. Careful attention to symptom chronology allowed the respiratory illness to be treated, while the delayed motor milestones, progressive proximal weakness, calf pseudohypertrophy, and Gowers maneuver prompted a separate neuromuscular evaluation. This dual-track approach is particularly important when an acute presentation risks overshadowing longstanding abnormalities that require independent investigation.

The markedly elevated serum CK level was the principal biochemical finding that strengthened suspicion of muscular dystrophy. A concentration of 8,500 U/L, approximately 34 times the upper reference limit, indicated substantial skeletal muscle injury but was not independently specific for DMD. Its diagnostic significance arose from its concordance with delayed motor development, progressive proximal weakness, calf pseudohypertrophy, and Gowers maneuver. The mild elevations in aspartate aminotransferase and alanine aminotransferase levels were also compatible with muscle-derived enzyme release. Transaminase elevations are well described in dystrophinopathies and may prompt unnecessary hepatic investigations when the possibility of skeletal muscle disease is overlooked [7]. Accordingly, this case highlights the potential value of early serum CK measurement in boys presenting with otherwise unexplained motor delay or proximal weakness, particularly when elevated transaminases occur without other evidence of hepatobiliary disease.

Molecular testing identified a pathogenic hemizygous deletion of exons 45-50 of the *DMD *gene that was predicted to disrupt the open reading frame. In conjunction with the clinical and biochemical findings, this result established the diagnosis of DMD and obviated the need for muscle biopsy in this patient. Current molecular diagnostic guidance recommends deletion and duplication analysis as a first-line component of genetic testing because exon-level copy-number variants account for a substantial proportion of dystrophinopathies [3]. Identification of the specific variant also informs carrier testing, reproductive counseling, and assessment for genotype-specific therapy. Deletion of exons 45-50 is potentially amenable to exon 51 skipping, which is intended to restore the translational reading frame and permit production of a shorter dystrophin protein. Clinical studies have reported dystrophin production with eteplirsen in patients with exon 51 skip-amenable DMD variants, although the clinical significance of these findings remains subject to the limitations of the available evidence [8]. Treatment suitability therefore depends on confirmation of the molecular result, the patient’s clinical and functional status, applicable regulatory criteria, local availability, and informed family preference. Genetic amenability alone should not be interpreted as evidence of assured clinical benefit.

Recognition of DMD at 11 years warrants timely multidisciplinary assessment because progressive skeletal muscle involvement may be accompanied by clinically silent cardiac and respiratory complications. Recommended care includes neuromuscular and functional assessment, consideration of corticosteroid therapy, physical therapy and contracture prevention, nutritional evaluation, and structured cardiac and respiratory surveillance [1,2]. For an ambulant child, a validated instrument such as the North Star Ambulatory Assessment can provide a standardized baseline of motor performance and facilitate longitudinal monitoring beyond isolated manual muscle-strength measurements [9]. In this patient, hypoxemia occurred during an acute respiratory illness; in the absence of pulmonary function testing, blood gas analysis, or sleep evaluation, the contribution of DMD-related respiratory muscle weakness to the hypoxemia cannot be determined. Important limitations of this report include the incomplete early motor history, absence of a standardized functional assessment, limited characterization of the respiratory infection and skin lesions, and lack of detailed longitudinal functional, cardiac, and respiratory outcomes. Despite these limitations, the case illustrates the value of investigating longstanding motor abnormalities alongside an acute illness, particularly when a focused neuromuscular examination and markedly elevated serum CK level suggest an underlying neuromuscular disorder.

## Conclusions

This case demonstrates that an acute respiratory illness may bring a previously unrecognized neuromuscular disorder to clinical attention. In a boy with delayed motor milestones, progressive proximal weakness, calf pseudohypertrophy, or difficulty rising, focused neuromuscular examination and serum CK measurement may help identify an underlying dystrophinopathy despite a competing acute presentation. Molecular testing can confirm the underlying dystrophinopathy, inform family counseling, and guide assessment of potential eligibility for genotype-specific treatment. The central message of this case is that acute and chronic findings should be evaluated in parallel, as recognition of longstanding motor abnormalities may help identify previously unrecognized neuromuscular disease and facilitate timely multidisciplinary evaluation.
